# Subjective well-being of left-behind children: a cross-sectional study in a rural area of eastern China

**DOI:** 10.1186/s13034-020-00333-7

**Published:** 2020-06-10

**Authors:** Lihong Ye, Yu Qian, Shuyang Meng, Ding Ye, Chao Rong, Eric E. Vandenhouten, Fangyuan Jing, Yingying Mao

**Affiliations:** 1 School of Public Health, Zhejiang Chinese Medical University, Hangzhou, 310053 China; 2 The Fourth College of Clinical Medicine, Zhejiang Chinese Medical University, Hangzhou, 310053 China; 3 School of Humanities and Management, Zhejiang Chinese Medical University, Hangzhou, 310053 China; 4 International Education College, Zhejiang Chinese Medical University, Hangzhou, 310053 China; 5 School of Public Health, Hangzhou Medical College, Hangzhou, 310053 China; 6 Department of Epidemiology and Biostatistics, School of Public Health, Zhejiang Chinese Medical University, 548 Binwen Road, Hangzhou, 310053 China; 7 Department of Epidemiology and Biostatistics, School of Public Health, Hangzhou Medical College, 481 Binwen Road, Hangzhou, 310053 China

**Keywords:** Left-behind children, Parent–child communication, Rural-to-urban migration, Subjective well-being

## Abstract

**Purpose:**

Psychological well-beings of left-behind children (LBC) in rural areas of China remain under-studied. In this cross-sectional study, we aimed to explore the subjective well-being (SWB) in LBC and its associated factors in a rural area in eastern China.

**Methods:**

Stratified random cluster sampling was used to select middle school and high school students in Qingyuan County of Zhejiang Province. Relevant information including sociodemographic characteristics was collected from each participant using an organized questionnaire. SWB was measured using the modified scale developed for Chinese adolescents. Univariable and multivariable regression analyses were performed using R version 3.3.0.

**Results:**

A total of 1086 children were recruited and examined in the current analysis, with 365 (33.61%) being left-behind. Compared with non-left-behind children (NLBC), LBC had significantly lower scores in family satisfaction (*P *= 0.003) and environment satisfaction (*P *= 0.020). Multivariable regression analysis uncovered that frequent parent–child communication was associated with high positive affect (*P *= 0.003) and life satisfaction (*P *< 0.001), and the type of caregivers was associated with negative affect among LBC (*P *= 0.037).

**Conclusions:**

Our results suggest SWB was lower in LBC, and targeted interventions including strengthening parental-child communication should be developed and implemented to improve LBC’s SWB in rural areas of China.

## Background

With the rapid economic development and urbanization, increasing numbers of rural labors have migrated to urban areas in China in the past 40 years [1, 2]. Due to various reasons, a large number of children were forced to leave behind by their migrant parents for indefinite periods. Data from the sixth national census showed that about 22% of all children, with an estimated number of 61 million, were left-behind in China [3].

Given the constant development in the quantity of left-behind children (LBC), significant research has been conducted to evaluate the health outcomes of these children. Several studies have indicated that children with experience of parent–child separation are increasingly susceptible to mental health problems. For example, compared with non-left-behind children (NLBC), LBC were more inattentive [4] and had a higher risk of depression [5], anxiety [6] and behavioral problems [7].

Subjective well-being (SWB) is a comprehensive index which incorporates both reflective subjective judgments of life satisfaction, and emotional responses to ongoing life in terms of positive and pleasant emotions versus unpleasant and negative feelings [8]. These emotions can be measured through self-report rating scales. These self-report measures are considered to have good reliability (genuinely stable over brief timeframes when there is little change in life circumstances) [9]. Evidence from cross-sectional, longitudinal, and intervention studies have demonstrated that degrees of SWB are correlated with numerous indicators of socially esteemed achievement, including successful outcomes in work, relationship, and health [10]. Albeit existing literature of SWB in children is yet constrained, studies have suggested that parental migration might have a significant impact on children’s SWB. For example, some studies reported that children being separated from both parents due to work migration had lower levels of SWB, as compared with NLBC or children with just a single parent separated due to work migration [11, 12]. A cross-sectional study conducted in Henan province in central China found that LBC had lower levels of life satisfaction and more elevated negative affect [13]. In addition, a longitudinal study conducted in Guangxi Province of South China reported that LBC had lower levels of life satisfaction, school satisfaction, and happiness [14].

Zhejiang Province is a relatively affluent province located in the east of China. However, there are a large number of LBC in the remote underdeveloped mountainous areas of Zhejiang Province such as Qingyuan County. To date, no study has been conducted to investigate the psychological well-beings of LBC in Qingyuan County. Therefore, in the present study, we conducted a cross-sectional survey of middle school and high school students in Qingyuan County to investigate LBC’s SWB and its associated factors such as parental migration status and parent–child communication, so as to provide information for the development of targeted intervention strategies.

## Methods

### Study population

This was a cross-sectional investigation directed from August to September, 2017, in Qingyuan County of Zhejiang Province in southeast China. By utilizing stratified random cluster sampling method, students from two middle schools and two high schools were enrolled. LBC were defined as children under 18 years old that have been left behind at their original residence for at least 6 months while one or both parents migrated for work. Children were categorized into different groups as indicated by their current left-behind status including “father migrated”, “mother migrated”, “both migrated”, “past migrated”, and a reference group of children (NLBC, neither parent migrated). The survey was conducted by Zhejiang Chinese Medical University School of Public Health, in cooperation with local health and educational authorities. The study was approved by the Ethical Committee of Zhejiang Chinese Medical University.

### Data collection

Socio-demographic characteristics of study participants were gathered by using a structured questionnaire by trained investigators. For LBC, data in regards to parental migration status, type of caregivers, contact frequency between parent(s) and child, and frequency of parent(s) visiting child were additionally recorded.

The modified SWB scale was used to evaluate children’s SWB status [15]. Briefly, the SWB scale was developed by Huebner E in 1994, which included 40 items to measure satisfaction with family, peers, school, self, and living environment of children and adolescents in the United States [16]. The scale was modified by Zhang X for Chinese adolescents and was used to measure SWB in a large-scale cross-sectional study in China in 2003 [15]. The modified SWB scale consisted of two parts, (i) life satisfaction scale, which was assessed by 36 items, including family satisfaction, friendship satisfaction, academic satisfaction, school satisfaction, environmental satisfaction and freedom satisfaction. Higher scores indicated better satisfaction of life; (ii) happiness scale, which was evaluated by 14 items, including positive affect and negative affect. Higher score indicated more grounded positive or negative emotions. This scale was reported to have unwavering quality and validity in a survey of > 1000 students in China, with Cronbach’s α coefficient of 0.943, and Kaiser–Meyer–Olkin of 0.949 [15], and has been applied to assess SWB of children in several other studies conducted in China [17–19].

### Statistical Analyses

Statistical analyses were performed using R software version 3.3.0, unless otherwise noted. Continuous data were analyzed utilizing Student *t* test or one-way analysis of variance (ANOVA), while categorical data were analyzed using Pearson’s Chi square test. Multivariable regression models were used to assess factors potentially associated with SWB in LBC. Two-sided *P*-values less than 0.05 were considered statistically significant.

## Results

### The basic characteristics of the study participants

A total of 1086 children aged 12–18 years were incorporated into the present study, with 365 (33.6%) being left-behind. Among them, 142 (38.9%) children were classified as father-migrated, 14 (3.8%) as mother-migrated, 162 (44.4%) as both-migrated, and 47 (12.9%) as past-migrated. In terms of caregivers of these LBC, 162 (44.4%) were cared by single parent, 123 (33.7%) by grandparents, 49 (13.4%) by uncles and aunts, 12 (3.3%) by brothers/sisters, and 19 (5.2%) by themselves without anyone else to care for them.

Table 1 presents the basic characteristics of LBC and NLBC of our study. Briefly, the percentage of LBC was higher among middle school students than in high school students (*P *= 0.012), and the percentage of LBC with parents divorced or passed away was higher than that of NLBC (*P *= 0.002). No statistically significant differences in the distributions of sex, ethnicity, grade, family income and other characteristics such as being a class leader or not, were observed between LBC and NLBC.

**Table 1:** Characteristics of left-behind children (LBC) and non-left-behind children (NLBC)

Characteristics of children	LBC (n = 365)		NLBC (n = 721)		χ^2^	*P*
	N	%	N	%		
Sex						
Female	186	51.0	405	56.2	2.45	0.117
Male	179	49.0	316	43.8		
Ethnicity						
Han	358	98.1	700	97.1	0.60	0.439
Others	7	1.9	21	2.9		
Location						
Village	123	33.7	223	30.9	0.73	0.392
Town	242	66.3	498	69.1		
Type of school						
Middle school	181	49.6	298	41.3	6.37	0.012
High school	184	50.4	423	58.7		
Boarding student						
Yes	286	78.4	585	81.1	1.01	0.315
No	79	21.6	136	18.9		
Having sibling(s)						
Yes	92	25.2	164	22.8	0.68	0.409
No	273	74.8	557	77.3		
Parents divorced or passed away						
Yes	43	11.8	44	6.1	9.85	0.002
No	322	88.2	677	93.9		
Household income						
Low	69	18.9	119	16.5	3.25	0.197
Middle	275	75.3	574	79.6		
High	21	5.8	28	3.9		
Class leader						
Yes	127	34.8	274	38.0	0.94	0.333
No	238	65.2	447	62.0		
Self-evaluation of academic performances						
Bad	74	20.3	124	17.2	2.85	0.583
Below average	55	15.1	119	16.5		
Average	126	34.5	260	36.1		
Above average	94	25.8	176	24.4		
Good	16	4.4	42	5.8		

*LBC* left-behind children, *NLBC* non-left-behind children

### Comparison of subjective well-being between left-behind children and non left-behind children

Table 2 exhibits the SWB status in LBC and NLBC, respectively. The scores (mean ± standard deviation) of life satisfaction, positive affect, and negative affect in LBC and NLBC were 4.7 ± 0.9 vs. 4.7 ± 0.8, 3.4 ± 1.3 vs. 3.4 ± 1.3, and 2.3 ± 0.9 vs. 2.3 ± 0.9, respectively, which did not differ significantly between the two groups (*P *> 0.05). Nonetheless, as for the subscales of life satisfaction, LBC had significantly lower scores in environment satisfaction (4.8 ± 1.0 vs. 5.0 ± 0.9, *P *= 0.020) and family satisfaction (5.2 ± 1.3 vs. 5.5 ± 1.1, *P *= 0.003), as compared with NLBC.

**Table 2:** Differences in subjective well-being (SWB) between left-behind children (LBC) and non-left-behind children (NLBC)

SWB	LBC (n = 365)		NLBC (n = 721)		T	*P*
	Mean	SD	Mean	SD		
Life satisfaction scale	4.7	0.9	4.7	0.8	1.20	0.229
Family satisfaction	5.2	1.3	5.5	1.1	3.00	0.003
Friendship satisfaction	4.9	1.1	4.9	1.0	0.84	0.400
Academic satisfaction	3.7	1.2	3.6	1.2	-1.37	0.171
School satisfaction	4.5	1.3	4.5	1.3	-0.45	0.650
Freedom satisfaction	4.6	1.2	4.7	1.1	1.19	0.233
Environment satisfaction	4.8	1.0	5.0	0.9	2.33	0.020
Happiness scale						
Positive affect	3.4	1.3	3.4	1.3	0.13	0.896
Negative affect	2.3	0.9	2.3	0.9	1.22	0.224

*LBC* left-behind children, *NLBC* non-left-behind children, *SD* standard deviation, *SWB* subjective well-being

### The subjective well-being and correlated factors among left-behind children

We further analyzed the SWB status in LBC and its conceivably related variables. As appeared in Table 3, parent–child contact frequency was significantly associated with positive affect (*P *= 0.003) and life satisfaction (*P *< 0.001) among LBC. The positive affect in LBC that contacted with their parents every day scored the highest (3.6 ± 1.4), while those contacted less than once per month had the least scores of positive affect (3.0 ± 1.2). In like manner, LBC that contacting with their parents every day scored the most noteworthy in life satisfaction (5.0 ± 0.8), while those with the recurrence lower than once per month had the least life satisfaction scores (4.2 ± 0.9). Results from multivariable regression models suggested similar findings (Table 4). Contact frequency between parent(s) and child was significantly associated with positive affect (*P *= 0.003) and life satisfaction (*P *< 0.001). Frequency of parents visiting children was positively associated with life satisfaction (*P *= 0.028), though it did not remain statistically significant in the multivariable analysis (P = 0.189) after adjusted for sociodemographic factors, parental migration status, type of caregivers, and contact frequency between parent(s) and child.

**Table 3:** Subjective well-being (SWB) of left-behind children (LBC) with different characteristics

Characteristics	N	%	Positive affect			Negative affect			Life satisfaction		
			Mean	SD	*P*	Mean	SD	*P*	Mean	SD	*P*
Parental migration status											
Father migrated	142	38.9	3.6	1.3	0.216	2.3	0.9	0.192	4.6	0.8	0.268
Mother migrated	14	3.8	2.5	0.9		2.3	1.0		4.3	0.7	
Both migrated	162	44.4	3.2	1.2		2.3	0.9		4.7	0.9	
Past migrated	47	12.9	3.5	1.4		2.1	0.5		4.8	1.0	
Type of caregivers											
Single-parent	162	44.4	3.5	1.3	0.282	2.2	0.8	0.116	4.6	0.9	0.628
Grandparents	123	33.7	3.3	1.3		2.3	0.8		4.6	0.8	
Uncles/aunts	49	13.4	3.2	1.2		2.3	0.9		4.8	0.9	
Brothers/sisters	12	3.3	2.9	1.3		2.0	0.4		4.9	0.9	
By oneself	19	5.2	3.5	1.2		2.7	1.2		4.5	1.1	
Contact frequency with parents											
1 per 30 days or more	43	11.8	3.0	1.2	0.003	2.5	1.1	0.161	4.2	0.9	< 0.001
1 per 15–30 days	71	19.5	3.3	1.3		2.3	0.8		4.5	0.8	
1 per 4–7 days	107	29.3	3.2	1.2		2.2	0.8		4.6	0.8	
1 per 2–3 days	69	18.9	3.6	1.3		2.1	0.8		4.8	0.8	
Daily	75	20.6	3.6	1.4		2.3	0.9		5.0	0.8	
Frequency of parents visiting children											
≥ 1 year	32	8.8	3.5	1.5	0.125	2.3	1.0	0.495	4.5	1.0	0.028
Every 6 months to 1 year	69	18.9	3.1	1.1		2.2	0.9		4.6	1.0	
Every 3–6 months	91	24.9	3.3	1.1		2.4	0.8		4.6	0.8	
Every 1–3 months	62	17.0	3.4	1.4		2.3	0.8		4.5	0.9	
Every month	111	30.4	3.5	1.4		2.2	0.9		4.9	0.8	

*LBC* left-behind children, *SD* standard deviation, *SWB* subjective well-being

**Table 4:** Multivariable regression analysis of factors associated with subjective well-being (SWB) among left-behind Children (LBC)

Variables	β	SE	t	*P*
Positive affect				
(constant)	3.015	0.278	10.864	< 0.001
Parental migration status	− 0.092	0.015	− 1.413	0.158
Type of caregivers	− 0.032	0.066	− 0.488	0.626
Contact frequency with parents	0.161	0.054	2.982	0.003
Frequency of parents visiting children	0.032	0.052	0.619	0.537
Negative affect				
(constant)	2.423	0.188	12.896	< 0.001
Parental migration status	-0.079	0.044	− 1.806	0.072
Type of caregivers	0.093	0.044	2.094	0.037
Contact frequency with parents	-0.035	0.036	− 0.971	0.332
Frequency of parents visiting children	-0.014	0.035	− 0.406	0.685
Life satisfaction				
(constant)	3.912	0.185	21.101	< 0.001
Parental migration status	0.011	0.043	0.258	0.796
Type of caregivers	0.019	0.044	0.440	0.660
Contact frequency with parents	0.164	0.036	4.557	< 0.001
Frequency of parents visiting children	0.046	0.035	1.315	0.189

*LBC* left-behind children, *SE* standard error, *SWB* subjective well-being

We observed that children with mother migrating had the most minimal positive affect (2.5 ± 0.9) and life satisfaction scores (4.3 ± 0.7), though the association did not reach statistical significance (*P *> 0.05). Multivariable regression analyses suggested that type of caregivers was associated with negative affect (*P *= 0.037), but not associated with positive affect (*P *= 0.626) or life satisfaction (*P *= 0.660). Children whose caregivers were brothers/sisters had the most minimal scores in negative affect (2.0 ± 0.4), while children taking care of themselves had the highest score of negative affect (2.7 ± 1.2).

## Discussion

In the present study, we found that environment satisfaction and family satisfaction in LBC were altogether lower than that in NLBC in Qingyuan County of rural eastern China. Furthermore, frequent contact between parents and children was related with high positive affect and life satisfaction among LBC.

Several studies have documented the negative impact of parental migration on children’s SWB status. A previous study conducted in African nations indicated that children with parents migrating had more unfortunate psychological well-being than children living with both parents [11]. Another cross-sectional study conducted in Henan province in central China likewise detailed that LBC had lower levels of life satisfaction and more elevated negative affect [13]. In addition, a longitudinal study including 897 rural children found that left-behind children revealed lower levels of life satisfaction, school satisfaction, and happiness [14]. Though we did not observe statistically significant differences in positive affect and negative affect between LBC and NLBC in Qingyuan County in the present study, we found that LBC had statistically significant lower scores in environment satisfaction and family satisfaction. Our discoveries were consistent with a previous cross-sectional study conducted in Jiangxi Province in China, which reported that children with one or both parents migrating had lower scores in environment satisfaction compared with NLBC [17].

A previous cross-sectional study that was conducted in Guangxi province in south China found that children with both parent migrating had lower scores of life satisfaction, compared with children with single parent migrating [20]. Though we did not find a statistically significant association between parental migrating status and SWB in LBC, our analyses suggested that children with mother migrating had the most reduced scores of SWB, compared with other types of parental migration status. It is possible that due to the work migration of the mother, the children cared for only by their fathers and brothers or sisters might receive inadequate emotional support. Consequently, children with mothers who leave for work migration for extended periods of time may require more attentions from their local neighborhood and the general public to improve their SWB status. In terms of the type of caregivers, we found that LBC whose caregivers were brothers/sisters had the most minimal scores in negative affect, while those taking care by themselves had the highest score of negative affect. This may because that brothers/sisters belonging to the same generation share similar emotional experience and could understand each other more, while those taking care of themselves may experience more negative feelings of lonely and abandoned [21].

In the present investigation, we found that parent–child communication was correlated with positive affect and life satisfaction of LBC. Similarly, as the study findings conducted by Su et al. [20] likewise revealed that children with more significant levels of parent–child communication had higher levels of life satisfaction, school satisfaction and happiness. These discoveries suggested that parent–child communication may play an important role in child development with work migration parenting.

There are strengths as well as limitations to the present study. To the best of our knowledge, this is the first principle study examining SWB and its associated factors among LBC in rural areas of eastern China. In any case, due to the cross-sectional design, we were unable to estimate the causal impact of parental migration on children’s SWB. Subsequently, further studies with a longitudinal design are warranted to surmise the causal factors related with SWB [22]. In addition, since the current study only included children from middle schools and high schools, our results may not necessarily apply to other age groups. Furthermore, as eastern China’s rural area is moderately affluent contrasted with other parts of rural China, the participants from rural areas in Qingyuan County might have characteristics different from populations in the central and western China rural areas, so the findings from this study might be limited when extrapolating to other populations.

## Conclusions

In summary, our study suggests that family satisfaction and environment satisfaction were lower in LBC, and parent–child communication is an important factor for the improvement of SWB in LBC. Targeted interventions including strengthening parental-child communication and paying special attention to LBC with mother migrating should be developed and implemented to improve LBC’s SWB in rural areas of China.

## Data Availability

The datasets analyzed in this study are available from the corresponding author on reasonable request.

## Abbreviations

ANOVA: Analysis of variance
LBC: Left-behind children
NLBC: Non-left-behind children
SE: Standard error
SWB: Subjective well-being
